# Composite structure of silken threads and a proteinaceous hydrogel which form the diving bell wall of the water spider *Agyroneta aquatica*

**DOI:** 10.1186/2193-1801-2-223

**Published:** 2013-05-16

**Authors:** Dietrich Neumann, Armin Kureck

**Affiliations:** Cologne Biocentre, University of Cologne, D 50674 Köln, Germany

**Keywords:** Water spider, Sheet-webs, Composite structure, Proteinaceous hydrogel, Diving bell construction

## Abstract

The unique ability of *Argyroneta aquatica* to form a diving bell web was re-examined using a new approach in a structurally simplified environment. The spiders generated sheet-webs from stiff, anchored threads and bundles of fine threads crossing each other, to which a hydrogel was added in several places. Due to the hydrophilic property of the web, small air bubbles could not pass this composite and remained perfectly spherical at the contact point. As revealed using Coomassie Brilliant Blue, the hydrogel and the silken threads are proteinaceous. The spider uses the web as a diving bell by transporting air bubbles to a small area underneath such a sheet-web, and by additional spinning activities. As revealed by light microscopy, the composite of threads and hydrogel is free of any meshes. In contrast, scanning electron microscopy shows only remnants of the hydrogel.

## Introduction

The palearctic diving bell spider, *Argyroneta aquatica* (Clerck 1757) (Araneae: Cybaeidae) is the only spider which lives and hunts submerged among water plants in ponds, ditches and lakes. Fine hydrophobic hairs on the abdomen (opisthosoma) hold a thin layer of air (a plastron) which enables the spider to breathe with its book lungs and tracheal system under water. In contrast to other spiders which hunt in an aquatic environment, this mainly nocturnal species is unique in spending its whole life under water. This is achieved by the use of a special web which is formed into a diving bell, the spider’s centre for rest and reproduction. By means of its plastron, the spider transports air from the water surface to a submersed horizontal sheet-web, thus forming and filling the bell (Braun 1931Wesenberg-Lund 1939Crome 1951Heinzberger 1974Masumoto et al. 1998;Nuridsany and Pérennou , Nuridsany and Pérennou Nuridsany and Pérennou 2004). Both sexes can make diving bells, but the sexes meet and copulate in the bell of the more sessile and smaller female; eggs are kept in a separate bell. This diving bell also functions as a physical gill (Schütz and Taborsky 2003;Schütz et al. , Schütz et al. Schütz et al. 2007Kehl and Dettner 2009Matthews and Seymour 2010Seymour and Hetz 2011). The fine structure of the diving bell wall was studied by De Bakker et al. (2006) and by Woermann (2010). On the basis of scanning electron microscopy studies, De Bakker et al. (2006) described different sizes of silken threads which form the diving bell wall and noticed *“that a kind of film was present, draped over and woven between the strands. It is not certain whether this film is produced by the spider (serving probably as a water repellent layer) or deposited by other aquatic organisms”* (p.139). The aim of the present study was to clarify the origin and role of this film.

## Material and methods

Adult specimens of *Argyroneta aquatica* were collected from a garden pond near Cologne and from a densely populated location in Upper Franconia.

**Figure 1:**
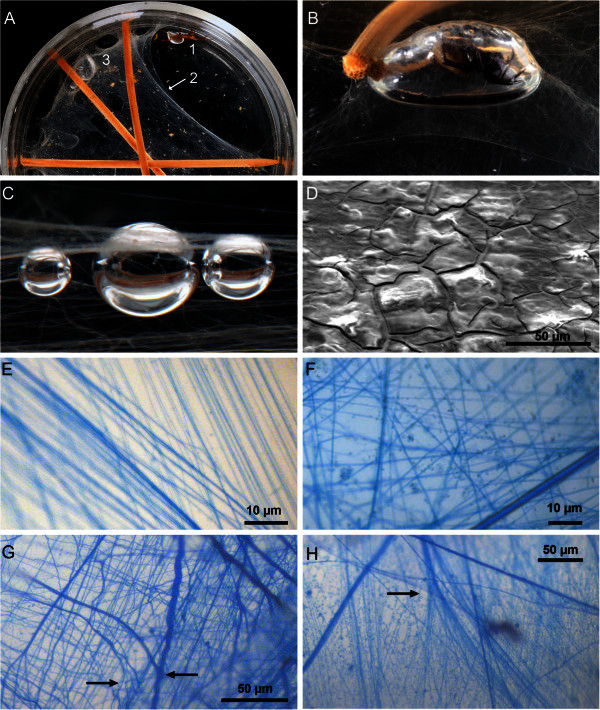
**Morphological properties of*Argyroneta*webs. A**: Top view of the experimental equipment for keeping water spiders. 1. specimen with its plastron, 2. anchor threads (see arrow) and various layers of sheet-web, 3. small, new diving bell and tunnels. **B**. Side view of the air volume of a one-night-old diving bell (max. width 20 mm) set beneath a slightly raised sheet-web (locally restricted by stronger threads anchored on both glass wall and sticks). The spider is sitting just inside its bell. **C**. Side view of three air bubbles (Ø 1.3–2.6 mm) experimentally placed using a micropipette beneath a sheet-web (the surface of the sheet-web is not visible over the largest air bubble due to its hyaline and thin properties). **D**. Surface of a sheet-web during early evacuation in a Fei Quanta FEG 250 SEM after it was exposed to air and mounted on an adhesive carbon tab. Its smooth surface has just become cracked. **E-H**. Threads and hydrogel of both sheet-web and diving bell wall scanned with a Zeiss Axioplan microscope after Coomassie Blue staining. **E**. Area of a one-night-old sheet-web with only two slightly crossing bundles of silk threads, still without any hydrogel in between. **F**. Area of a completed sheet-web with both strong and very thin hydrophilic silk threads embedded in a proteinaceous hydrogel. **G**. Complex structure of a one night-old diving bell wall with crossing strong and fine threads embedded in bluish hydrogel (the scan was processed by a digital contrasting programme). Additionally, one can detect places at which crossing threads are linked together (see arrows). **H**. Structure of a two-night-old diving bell wall with a strong silk thread splitting into thin threads (or vice versa: fine threads combining to a strong thread?).

As it was difficult to see details of the diving bell construction between aquatic plants, these spiders were observed under simplified structural conditions. Single individuals were kept in glass vessels (Ø 12 cm, with about 400 cm^3^ tap-water) equipped with three bamboo skewers of equal length which were horizontally clamped between the walls about 2–3 cm beneath the surface (Figure 1A). Water temperature was 21°C and the laboratory had natural daylight between September and May and was dimly lit by the campus lighting at night. Exposed to this situation, some spiders clung to the bamboo sticks and remained inactive until night. Others were immediately active and started spinning.

The spiders were fed every 2–3 days with *Daphnia magna* or *Gammarus* sp. One female successfully reproduced within its diving bell, resulting in about fifty offspring during the winter months.

Light-microscopic images of wet and air-dried sheet-webs and diving bells (Figure 1E-H) were scanned with a ZEISS Axiophot2, combined with the digital image processing system Axiovision. Scanning electron microscope (SEM) pictures were taken in two different ways: with a Hitachi S 520 after gold-coating of dried samples *en vacuo* (Inst. Physical Chemistry), and with a Fei Quanta FEG 250 (Zoological Institute) in which the wet sheet-webs could be directly exposed without previous fixation or coating.

Small gas bubbles released below the sheet-web from a fine Hamilton syringe were used to test the permeability of the new web.

The proteinaceous property of both the threads and the hydrogel of fresh, wet sheet-webs and diving bells was tested with a specific Coomassie stain. After fixation in a mixture of 10% AcOH and 50% MeOH equivalents for at least 6 hrs, the material was stained in this liquid with 0.2% ‘Coomassie Brilliant Blue R-250′ in 0.12 mol AcOH in 50% MeOH equivalent for at least 30 min, followed by a decolouration over two steps in 10% AcOH in 20% MeOH equivalents for about 8 hrs. Finally, the sample was transferred into distilled water (Bradford 1976).

## Results

### Construction of sheet-webs

During the first night all spiders produced a horizontal web of variable size. Figure 1A shows a two-night-old web with a small diving bell and tunnel-like pathways near the glass wall.

Spiders which had already started to spin in daylight began with single, tightly stretched anchor threads (thickness up to 1.8 μm). These were set between crossing sticks or between a stick and the glass wall. The anchor threads were fixed by a small blob of ‘cement’ visible on the glass wall. The spiders continued to add bundles of smaller, parallel threads (0.5–1.0 μm thick) between these strong threads, or between the anchor threads and the sticks (Figure 1E). In this way, a coarse scaffold for a horizontal sheet-web was formed within 2–4 hours. In most cases it was still permeable for small gas bubbles (1–2 mm in diameter) released below the web by a micropipette. However, during the first night, such web compartments were functionally completed far enough that no bubbles could pass (Figure 1C).

### Fine structure of horizontal sheet-webs and diving bell walls

**Figure 2:**
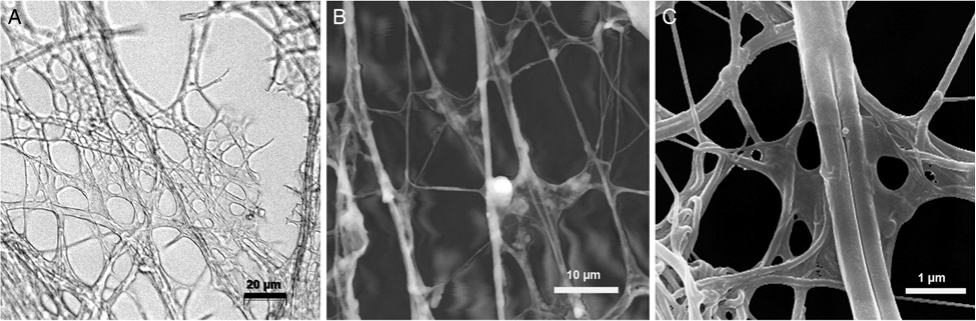
**Dried structure of diving bell walls, demonstrating various types of silk threads and remains of the proteinaceous hydrogel at crossover points. A**: Air-dried sheet-web stretched out on a PVC net and examined under a light microscope (no fixation, no staining). **B**: SEM-photo of a drying sheet-web during evacuation, examined under a Fei Quanta FEG 250. **C**: SEM-photo of a diving bell wall by a Hitachi S 520, after gold coating *en vacuo*.

Completed sheet-webs appeared slightly opaque and jelly-like under a stereo microscope, and no mesh-structures were seen. If a new wet sheet-web was exposed to the slowly increasing vacuum of a Fei Quanta FEG scanning electron microscope, its smooth surface became cracked due to the removal of water (Figure 1D). The water content of the hydrogel was obviously higher than that of the threads: the smooth surface first broke up along an interphase of hydrogel and silk threads, similarly to how mud cracks when it dries. In any case, sheet-webs were immediately deformed when exposed to air or to a vacuum (Figure 2). New diving bell walls showed the same properties.

Sheet-webs and diving bells stained with Coomassie Blue (which binds to proteins) showed three different components, see following Table 1.

**Table 1:** **Components of sheet-webs and diving bells**

Type	Colour	Thickness	Properties	See
Single threads	blue	variable	crossing each other	Figure 1 F-H
Flat bundles	blue	200–400 μm	crossing other strips, with single threads Ø < 1μm	Figure 1 G, Figure 1 E-G
Gel-like mass	bluish	homogenous	embedding all threads	Figure 1 F-H

There was no structural difference between a completed sheet-web and a new diving bell wall without further reinforcements. In both structures, a few anchor threads were sometimes split into fine threads (Figure 1H, arrow).

### Wetting property of sheet-webs and diving bell wall

Figure 1C shows three small air bubbles below a sheet-web and in contact with its surface. The bubbles have a spherical form, indicating that the value of the effective contact angle θ_e_ at the point of the three phase contact water/air/sheet-web is close to zero. Similar characteristics are found in diving bells after the water spider has brought the first air bubbles underneath a small sheet-web during the very early stages of the construction.

### Extension and layer thickness

A diving bell could be formed as soon as the horizontal sheet-web was filled with hydrogel. The air volume was enlarged step by step, whereby the anchored sheet-web lifted slightly due to the buoyancy of the air underneath. After two nights the bell was big enough for the spider to rest in (Figure 1B). Further spinning activities at its inner side reinforced the wall. Anchor threads and part of the remaining sheet-web (see Figure 1B) stabilized the growing bell.

The sheet-webs in our experiments were considerably larger than those found under more natural conditions, in some cases up to about 80 cm^2^ (Figure 1A). This demonstrates the capacity of the spiders’ silk glands. The thickness of these sheet-webs could not be assessed because various layers of threads were superimposed. The walls of a new diving bell (mounted by a polyethylene net with 0.8 mm mesh size on a microscope slide in water) were about 40 μm thick (n = 15). However, such a preparation did not represent a functionally intact diving bell wall, since the wall was not stretched by the internal pressure of the air volume. The real layer thickness of the diving bell’s wall should be far less, presumably about 10 μm, even if strengthened by further spinning secretions.

### Fine structure of dry sheet-webs and diving bells

Sheet-webs and the walls of diving bells quickly lose their hydrogel-like appearance and become perforated when they dehydrate (Figure 2, left). The hydrogel shrinks, but membranous-like pieces remain between crossing threads. Thin threads can break. Attempts to rehydrate a dehydrated web failed.

An even stronger drying effect occurred during the gold coating process (for taking SEM-images with a Hitachi model). Figure 2 (right) shows the framework of various dried threads and the membranous remains of the dried hydrogel between the thin threads. Without gold coating, one could continuously follow this drying process in a Fei Quanta FEG Scanning microscope (Figure 2, middle). The linear threads became increasingly thin. When a thread tore, the ends were pulled back towards their points of attachment and swelled to several times their extended diameter, demonstrating their elasticity. The strong anchor threads generally shrank to about 0.5 μm in diameter, while the smaller ones shrank to 0.14–0.23 μm before they tore.

## Discussion

The spherical form of air bubbles caught under a sheet-web or inside a new bell indicated that the value of the effective contact angle θ_e_ at the point of the three phase contact water/air/sheet-web was close to zero, confirming a physical argument according to which the surface of the wall must be hydrophilic (Woermann 2010).

The proteinaceous hydrogel, which is described here for the first time, corresponds in its properties to an ensemble of hydrophilic macromolecules forming a network in water. We assume that the solid fragments which can be seen in the SEM-pictures published by De Bakker et al. (2006) are remains of this hydrophilic hydrogel component. Several questions about the hydrogel remain open for further investigations, foremost concerning its chemical constitution. Is it some liquid silk, as primarily produced in the spinning apparatus glands, but without further structural differentiation? Or is it a glycoprotein secreted by a distinct type of spinneret gland? The pronounced hydrophilicity of both the silk threads and the hydrogel should also be the subject of further investigation. The new method of keeping the spiders offers the possibility of gaining sufficient amounts of the secreted products.

*Argyroneta aquatica* has three pairs of spinnerets with a large number of spigots (Foelix 1996). One has to assume that all components of the composite are products of the silk glands. However, the relationships between the components of the composite and distinct spigots or silk glands have not yet been analysed, nor has the mechanism by which the fine threads become twisted to a strong anchor thread been investigated yet (or vice versa).

The diving bell has to withstand a hydrostatic pressure difference corresponding to the pressure exerted by a vertical water column of up to several decimetres, and its wall must remain expandable. At the same time, it has the function of a physical gill. The observed combination of threads in the hydrogel which connects them like a flexible binder clearly meets this requirement. During the stepwise enlargement of the diving bell, *Argyroneta aquatica* reinforces it by further spinning on its inner wall. Since all components of the composite are hydrophilic, the adhesive forces between threads and hydrogel contribute to the stability of the bell wall, and the hydrogel can patch up areas of the wall and improve its stability. The adhesive forces between the components are documented by SEM-pictures taken during the drying process (Figure 2, middle). It is possible that the hydrogel also lowers the hydrodynamic permeability of the wall, thus suppressing a convective volume flow across the wall, and may retard the gas exchange between the air volume of the diving bell and the external water. Hence, the air-water interface at the open bottom of the diving bell might be important for the overall balance of the gas transfer, as recorded by Seymour and Hetz (2011). A retarded gas transfer via the bell wall may be an advantage for the resting spider when low pO_2−_values occur on warm summer nights and could reduce the number of the spiders’ trips to the surface to collect fresh air.
